# eHealth tools to improve the health-related quality of life of family caregivers of cancer patients in developing countries: a systematic review protocol

**DOI:** 10.3332/ecancer.2025.2015

**Published:** 2025-10-14

**Authors:** Israel Gabriel, Andy Emmanuel, P Pratitha, Divitha Subramanian, Thilaka Chandanee, Fariba Hosseinisazi

**Affiliations:** 1 Institute of Health and Management, Sydney 2150, Australia; 2 Community Education Instructor, Queensland Ambulance Service, Brisbane, QLD 4207, Australia; a https://orcid.org/0000-0002-5663-450X; b https://orcid.org/0000-0002-8563-4306

**Keywords:** e-health, telehealth, telemedicine, quality of life, well-being, family caregivers of cancer patient, informal caregivers, developing countries

## Abstract

**Background:**

ehealth improves the health-related quality of life for cancer patients and their families by providing easier access to medical information, promoting self-management, and providing personalised care through digital platforms. However, there is still a dearth of comprehensive understanding of their influences in developing countries.

**Objectives:**

To identify several ehealth interventions accessible to family caregivers of people with cancer in developing countries and to assess the impact of these interventions on their health-related quality of life.

**Methods:**

The review will adhere to the Preferred Reporting Items for Systematic Reviews and Meta-Analysis Protocols. Health databases and search engines, including PubMed, Medline via Ovid, Embase via Ovid, and CINAHL via EBSCOhost, will be used. We will include quantitative or mixed method research evaluating e-health, with a particular emphasis on the health-related quality of life of family caregivers of cancer patients. Studies from developing countries, including peer-reviewed journals and grey literature, will be considered without regard to publication date.

The study selection process involves screening titles and abstracts for relevance, then, doing a full-text assessment against the inclusion criteria. The Cochrane Risk of Bias tool for randomised controlled trials and ROBINS-I V2 for non-randomised studies will be employed to assess the quality of the included studies. The A Measurement Tool to Assess Systematic Reviews will assess the quality of this systematic review.

**Implications:**

This review offers a thorough and impartial summary of current research on e-health tools and their impact on the health-related quality of life of family caregivers of cancer patients. It seeks to inform evidence-based decision-making across healthcare, policy development, and research design by identifying knowledge gaps, emphasising areas requiring further investigation, and steering future research.

**Systematic review registration:**

PROSPERO CRD42024622302

https://www.crd.york.ac.uk/PROSPERO/view/CRD42024622302

## Background

Cancer constitutes a significant global health burden [1]. In 2022, the worldwide cancer burden was at 20 million individuals, resulting in 9.7 million cancer-related deaths [2]. By 2050, almost 35 million new cancer cases are anticipated, reflecting a significant 77% rise from the estimated 20 million cases recorded in 2022 [3]. The population's growth and the number of people reaching advanced ages, along with shifts in the distribution and prevalence of the key risk factors for cancer, many of which are linked to socioeconomic development, are the main causes of the increase in new cancer cases [4]. Notwithstanding these challenges, the population of cancer survivors is steadily increasing, with improvements in cancer survival rates attributable to advancements in technology and pharmacological research in cancer treatment [5, 6]. A considerable number of individuals who have survived cancer are required to navigate the physical effects of the disease and its treatment, which may result in functional and cognitive challenges, alongside a range of psychological, social, spiritual and economic consequences [7–9].

During cancer treatment, patients become more reliant on others, have diminished performance and face heightened physical and psychological challenges [10]. Consequently, family caregivers are an integral part of the caregiving continuum, significantly contributing to the support of these patients [11]. Family caregivers often provide extended, intensive personal care without respite, compensation or external support. Providing care and support during this period can be a difficult task [12]. Many family caregivers prioritise the needs and emotions of their loved ones with cancer over their own [13]. Physical care, assistance with daily tasks, medication administration, transportation, emotional support, household chores and companionship are just a few of the responsibilities they do [14]. Likewise, family caregivers engage in care and treatment decisions while monitoring treatment side effects and symptoms with diligence [15]. Nonetheless, providing comprehensive care and support to family members may adversely affect the psychological, social, physical and spiritual well-being and overall health-related quality of life (HRQoL) of family caregivers, potentially endangering their health [16, 17]. HRQoL is a subjective assessment that encapsulates an individual's perceptions and emotions regarding their health and its impact on daily living. It is a multifaceted concept that evaluates the impact of health, disease or injury on an individual's overall well-being and life satisfaction. It pertains not just to the lack of sickness but also to physical, mental and social well-being [18].

There has been a lot of interest in developing focused interventions to meet the needs of cancer patients' family caregivers [16, 19–21]. A substantial amount of research has recorded the effect of interventions on the HRQoL of family caregivers of people living with cancer [20–23]. For example, Chow *et al* [22] conducted a systematic review and meta-analysis to investigate the effectiveness of interventions designed to support caregivers of patients with advanced cancer, focusing on caregivers' QoL and mental health outcomes. The review encompassed 56 articles that reported on randomised controlled trials (RCTs), mostly involving psychoeducational or problem-solving interventions. The interventions demonstrated significant effects on overall QoL, mental well-being, anxiety and depression in comparison to standard care. Ahn *et al* [20] similarly reported in a systematic review encompassing 11 studies, with interventions categorised as psychological, educational or a combination of both. Most interventions had statistically significant outcomes in reducing psychological distress and caregiving burden, while improving QoL, self-efficacy and caregiving competence. Conversely, Kalyani *et al* [23], in their systematic review and meta-analysis involving eight studies to examine the effects of psychosocial interventions on the QoL of cancer caregivers, reported that while these interventions were found to improve caregivers' QoL, but not statistically significantly.

eHealth is another significant resource for meeting the care needs of cancer patients' family caregivers, particularly in developed countries with well-established and stable healthcare systems facilitated by eHealth [16, 24, 25]. eHealth in oncology includes psychoeducational programs, remote symptom monitoring, self-care education, online peer support forums, physical activity tools and various other applications using digital platforms [14, 26]. eHealth may significantly improve healthcare delivery through prevention, early diagnosis, medication safety, treatment adherence by patients, guideline adherence by providers, medication safety, improved care coordination, documentation, data management and research, among others [27–29]. eHealth has the potential to enhance cancer care delivery and research, hence reducing cancer-related morbidity and mortality in developing countries, if it is embraced and effectively implemented throughout the cancer continuum [30]. However, this is not true in many developing countries, where eHealth has considerable gaps in acceptance and effectiveness [31]. These gaps include a lack of infrastructure, digital knowledge and resources, which impedes broad acceptance and equal access to eHealth technologies, as well as death of shortage of trained professionals, impede the integration of eHealth technology [32]. Moreover, there is a lack of published literature on the application of eHealth solutions in oncology in developing countries, particularly in African countries such as Nigeria, Ghana, Kenya and other sub-Saharan regions [33–36]. Most of the existing literature focuses on infectious diseases, including

HIV/AIDS, Malaria and Tuberculosis [37, 38]. Consequently, addressing these gaps is crucial for realising the potential of eHealth to improve cancer outcomes in developing countries. Thus, the purpose of this review was to identify eHealth interventions aimed at family caregivers of people living with cancer in developing countries and the effects on their HRQoL.

**Review question(s)**

1. What eHealth interventions are accessible for family caregivers of cancer patients in developing countries?
2. What is the effectiveness of these eHealth interventions in improving the HRQoL for this population?

## Methods

### Protocol and registration

The systematic review will follow the guidelines set by the Preferred Reporting Items for Systematic Reviews and Meta-Analysis Protocols (PRISMA-P) statement to ensure that the protocol is complete and meets high standards [39, 40] (Supplementary file 1). The methodological approach encompasses three key components: the search strategy, the selection of relevant articles and the critical evaluation of their methodological quality. The review protocol is prospectively registered with the International Prospective Register of Ongoing Systematic Reviews (PROSPERO) database (registration number CRD42024622302). https://www.crd.york.ac.uk/PROSPERO/view/CRD42024622302

### Eligibility criteria

The eligibility criteria for this review will be determined using the Population, Intervention, Comparison, Outcome (PICO). framework. We will include studies that (a) are RCTs or non-randomised trials with or without a control condition or observational studies; (b) target unpaid family caregivers of cancer patients aged 18 years and older; (c) comprise eHealth interventions, such as psychoeducational programmes, remote symptom monitoring, self-care education, online peer support forums, physical activity tools and other technology-based applications; (d) report quantitative outcomes of family caregivers of cancer patients, such as psychological functioning, HRQoL, physical health and general functioning and (e) studies undertaken in English and other languages, published in countries designated by the United Nations as developing, characterised by low living standards, inadequate infrastructure and a predominantly non-industrialised economy [35] (Table 1).

### Information sources

Health databases, specifically PubMed, Cochrane, Medline via Ovid, Embase via Ovid and CINAHL via EBSCOhost, will be meticulously searched for original articles. The search will cover scientific articles published in English and other languages from the inception of the databases until 2024. We will conduct manual searches of abstracts from the reference lists of pertinent articles to identify supplementary studies.

### Search strategy

The search strategy will seek to identify published studies. This review will employ a three-phase search strategy. First, the preliminary search of PubMed was conducted to establish a fundamental understanding of the research landscape on the subject. Additionally, facilitate the refining of review questions and identify essential terminology. The terminology found in the titles and abstracts of pertinent articles will be used to characterise the articles and along with controlled vocabulary, a full search strategy will be developed for the full review. The search strategy, encompassing all selected keywords and index terms, will be tailored for each health database and/or information source. A librarian contributed to the development of the initial search strategies and will participate in the final search strategy and the management of search results.

The second phase will involve a simultaneous search to enhance the sensitivity of the results, following the completion of all identified keywords and index terms. The four health databases that will be searched are PubMed, Medline via Ovid, Embase via Ovid and CINAHL via EBSCOhost. Additionally, the associated platforms will be searched.

The third phase of the search strategy involves the screening of the reference lists of all included sources, which will be examined for supplementary studies. This review will exclusively encompass quantitative and mixed-method studies. Studies published in languages other than English will not be included; however, all publication years will be included.

### Study records

#### Data management

We will use Covidence software to facilitate screening, data extraction, dispute resolution, author review and validation of data, development of data tables and creation of data files for future study use. Covidence is a leading tool for coordinating systematic reviews that is effectively utilised in various projects across several research fields, particularly healthcare [41].

#### Selection process

Records will be compiled and duplicates will be removed using EndNote 20 software. The data will be managed with Covidence software. The screening and selection procedure will adopt a three-phase approach to ensure precision and comprehensiveness.

A team of three reviewers will evaluate titles and abstracts using predefined inclusion and exclusion criteria. Two team members will independently evaluate titles and abstracts once all raters reach a 90% consensus.

**Figure 1.**
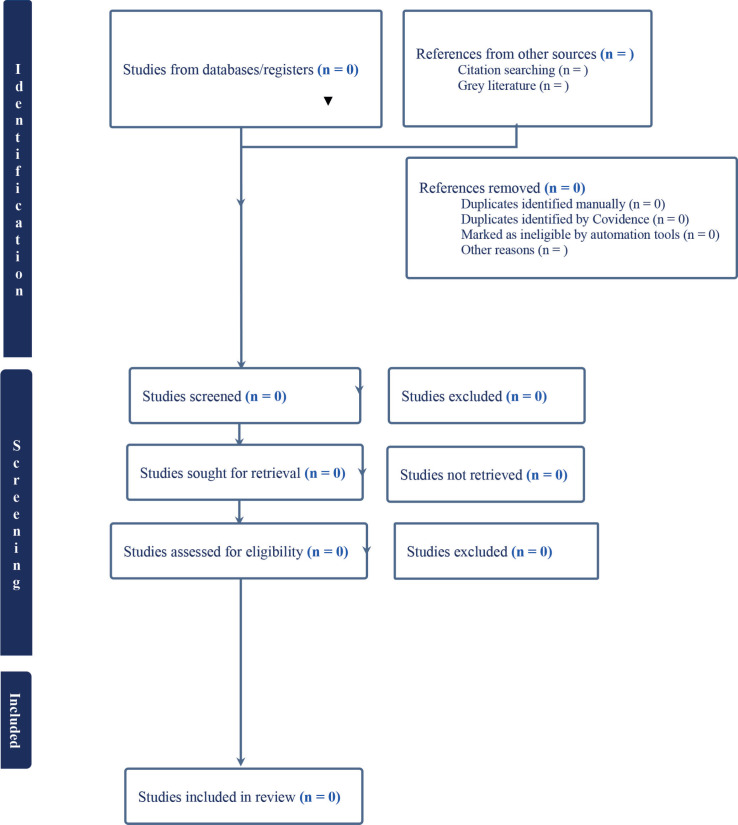
PRISMA flow diagram of study retrieval and inclusion process, adapted from (PRISMA, 2021).

The second phase is a comprehensive review of the full text of selected articles. Two independent reviewers will check the full text against the inclusion criteria and write down reasons for any articles they exclude, which will be carefully noted in the PRISMA-P flow diagram [42], as shown in Figure 1.

The third phase involves two independent reviewers deciding on the final inclusion of studies. We will address disagreements among reviewers either through discussion or by including additional team members. The results of the search and the studies that were included will be clearly explained in the systematic review and shown in a PRISMA 2020 flow diagram [40].

#### Data collection

Two independent reviewers will methodically retrieve data from the included studies using Covidence software. We intend to extract data regarding publication information, conceptualisation components, methodologies and outcomes. We will extract author names, year of publication, journal and, if applicable, funding source from publication information. We will extract information on the study questions or hypotheses, theoretical or conceptual frameworks and types of interventions within the components of conceptualisation. Regarding the methodology, we will extract the study design, study setting, sampling and recruitment processes and data collection methods.

For the outcomes, we will extract research findings on interventions and their impact on HRQoL. To facilitate a methodical and coordinated approach to data extraction, at least two authors will extract from each article to ensure consistency. The two authors shall meet to deliberate and assess the extracted data for any missing information or conflicting reports. Independent authors shall convene with extractors to attain a consensus regarding any discrepancies in information, should the primary reviewers be unable to reach a definitive conclusion.

#### Risk of bias in individual studies

Three authors will independently use the Cochrane Risk of Bias tool for RCTs [43] and ROBINS-I V2 for non-randomised studies [44]. The Cochrane Risk of Bias tool evaluates the randomisation process, potential biases, allocation concealment, outcome assessment, reporting of results and attrition rates. ROBINS-I V2 evaluates possible bias across multiple domains, including exposure/intervention assignment, confounding variables, selection bias, information bias and attrition bias.

#### Assessment of methodological quality

Four reviewers will independently evaluate the quality of systematic reviews using the A Measurement Tool to Assess Systematic Reviews (AMSTAR) tool [45]. AMSTAR is an 11-item tool widely regarded as one of the most effective tools for assessing the methodological quality of systematic reviews, demonstrating strong face and content validity. The instrument allows researchers to assess the quality of a systematic review by examining aspects such as the literature search strategy, study selection process and analysis methods, thereby helping to determine the reliability of its findings [46].

#### Data synthesis

The evidence will be described using a narrative approach. The methodological rigour of the included studies will be evaluated to ascertain the potential effect of biases on the results, utilising a standard evaluation technique, followed by a synthesis of their findings. Furthermore, all authors will review all included articles independently before integrating the reviews and arriving at a consensus where there is conflicting or opposing outcome. This review focusses on the following domains: author, year, setting, theoretical framework, kind of eHealth, purpose, research design, response rate, data collection, outcome measures and findings. The included studies' methodological rigour will be evaluated using a standard assessment tool to establish the extent to which biases may influence. The domains of interest in this review includes author, year, setting, theoretical framework, type of eHealth, purpose, study design, response rate data collection, outcome measures and findings.

Care will be taken to ensure that any overlap between the primary studies included in the systematic reviews is accounted for and considered when deriving conclusions from the evidence. The limitations of the data and gaps in the evidence will also be highlighted.

## Discussion

This systematic review will provide a thorough and effective review of the existing evidence regarding technology-based interventions aimed at enhancing HRQoL. The strengths of this systematic review will be found in its organised approach and extensive, comprehensive search strategy, quality assessment and data analysis. This approach will identify all relevant literature to assess the effectiveness of eHealth tools in enhancing the HRQoL for family caregivers of cancer patients in developing countries. This will guide future intervention policies and commissioning decisions. Furthermore, it will identify areas of weakness, inconsistency and gaps in the evidence base for eHealth tools aimed at enhancing HRQoL concerning secondary evidence, thereby guiding future research efforts. The work will emphasise both the areas where well-conducted systematic reviews have revealed weaknesses in the primary evidence, as well as the weaknesses of the secondary evidence, particularly regarding low-quality systematic reviews.

## Ethics and dissemination

Since this is a review of original studies previously approved by research ethics committees, it is unnecessary to submit the review to the assessment of ethical aspects involving human beings. The policies and practices of healthcare institutions and governmental health organisations may be influenced by the results of this study, which will be disseminated through peer-reviewed publications, webinars and conferences. Additionally, we will employ the data to create new studies that resolve the identified academic gaps.

## Conflicts of interest

No conflicts of interest.

## Funding

There is no funding for this review protocol.

## Figures and Tables

**Table 1.** Inclusion and exclusion criteria.

	Inclusion criteria	Exclusion criteria
Study design	Intervention studies RCTs, non-randomised trials with or without a control condition, and observational studies.	Unpublished studies Qualitative design only Non-English articles
Population	The family caregivers of people living with cancer	Studies that focused on family caregivers of chronic conditions other than cancer or the inability to access digital media.
Intervention	eHealth of any kind includes psychoeducational programmes, remote symptom monitoring, self-care education, online peer support forums, physical activity tools and various other applications using technology.	Any other interventions that did not include eHealth
Comparison	Studies will be included that involve comparison, such as RCTs and quasi-experimental designs, as well as studies without comparison, such as single-group and observational studies.	None
Outcomes	Psychological functioning, HRQoL, physical health or general functioning.	Did not measure psychological functioning, HRQoL, physical health or general functioning.

## Supplementary

**Supplementary file 1.** PRISMA-P 2015 checklist: recommended items to address in a systematic review protocol*.

Section and topic	Item No	Checklist item
**Administrative information**		
Title:		
Identification	1a	Identify the report as a protocol of a systematic review
Update	1b	If the protocol is for an update of a previous systematic review, identify as such
Registration	2	If registered, provide the name of the registry (such as PROSPERO) and registration number
Authors:		
Contact	3a	Provide name, institutional affiliation, e-mail address of all protocol authors; provide physical mailing address of corresponding author
Contributions	3b	Describe contributions of protocol authors and identify the guarantor of the review
Amendments	4	If the protocol represents an amendment of a previously completed or published protocol, identify as such and list changes; otherwise, state plan for documenting important protocol amendments
Support:		
Sources	5a	Indicate sources of financial or other support for the review
Sponsor	5b	Provide name for the review funder and/or sponsor
Role of sponsor or funder	5c	Describe roles of funder(s), sponsor(s), and/or institution(s), if any, in developing the protocol
**Introduction**		
Rationale	6	Describe the rationale for the review in the context of what is already known
Objectives	7	Provide an explicit statement of the question(s) the review will address with reference to PICO
**Methods**		
Eligibility criteria	8	Specify the study characteristics (such as PICO, study design, setting, time frame) and report characteristics (such as years considered, language, publication status) to be used as criteria for eligibility for the review
Information sources	9	Describe all intended information sources (such as electronic databases, contact with study authors, trial registers or other grey literature sources) with planned dates of coverage
Search strategy	10	Present draft of search strategy to be used for at least one electronic database, including planned limits, such that it could be repeated
Study records:		
Data management	11a	Describe the mechanism(s) that will be used to manage records and data throughout the review
Selection process	11b	State the process that will be used for selecting studies (such as two independent reviewers) through each phase of the review (that is, screening, eligibility and inclusion in meta-analysis)
Data collection process	11c	Describe planned method of extracting data from reports (such as piloting forms, done independently, in duplicate), any processes for obtaining and confirming data from investigators
Data items	12	List and define all variables for which data will be sought (such as PICO items, funding sources), any pre-planned data assumptions and simplifications
Outcomes and prioritization	13	List and define all outcomes for which data will be sought, including prioritization of main and additional outcomes, with rationale
Risk of bias in individual studies	14	Describe anticipated methods for assessing risk of bias of individual studies, including whether this will be done at the outcome or study level, or both; state how this information will be used in data synthesis
Data synthesis	15a	Describe criteria under which study data will be quantitatively synthesised
	15b	If data are appropriate for quantitative synthesis, describe planned summary measures, methods of handling data and methods of combining data from studies, including any planned exploration of consistency (such as I2, Kendall’s τ)
	15c	Describe any proposed additional analyses (such as sensitivity or subgroup analyses, meta-regression)
	15d	If quantitative synthesis is not appropriate, describe the type of summary planned
Meta-bias(es)	16	Specify any planned assessment of meta-bias(es) (such as publication bias across studies, selective reporting within studies)
Confidence in cumulative evidence	17	Describe how the strength of the body of evidence will be assessed (such as GRADE)

* It is strongly recommended that this checklist be read in conjunction with the PRISMA-P Explanation and Elaboration (cite when available) for important clarification on the items. Amendments to a review protocol should be tracked and dated. The copyright for PRISMA-P (including checklist) is held by the PRISMA-P Group and is distributed under a Creative Commons Attribution Licence 4.0

From: Shamseer L, Moher D, and Clarke M, *et al* (2015) **Preferred reporting items for systematic review and meta-analysis protocols (PRISMA-P) 2015: elaboration and explanation** *BMJ* **349**(jan02 1) g7647
